# A Method to Calculate the Support Length of Beams Resting on Masonry Walls

**DOI:** 10.3390/ma14237131

**Published:** 2021-11-23

**Authors:** Marco Andrea Pisani, Massimiliano Bocciarelli, Tommaso D’Antino

**Affiliations:** Department of Architecture, Built Environment, and Construction Engineering, Politecnico di Milano, 20133 Milan, Italy; marcoandrea.pisani@polimi.it (M.A.P.); massimiliano.bocciarelli@polimi.it (M.B.)

**Keywords:** masonry, design, FEM, support condition, analytical procedure

## Abstract

Rehabilitation, strengthening, and retrofitting of existing masonry buildings represent an important challenge for the construction engineering field. Often, slab strengthening/retrofitting is performed by replacing existing timber and steel beams or by adding new beams to improve the slab load-carrying capacity. The computation of the stresses at the beam–masonry interface (i.e., the contact pressure) is crucial to properly design the beam support length, preventing local failure of masonry and beam. This paper presents a simple analytical procedure to compute the contact pressure at the beam–masonry interface. The analytical procedure is validated by comparison between analytical and corresponding numerical results obtained by finite element modeling. Different types of beam (solid and laminated timber beams and steel beams) were considered, as well as different support conditions (simply resting on the wall considering different support lengths or fully embedded). The results obtained show that the method proposed is simple and reliable, which makes it suitable for professional practice.

## 1. Introduction

Structural rehabilitation of historical masonry buildings often involve replacement or strengthening of existing slabs. In case replacement is needed, a traditional slab made of timber beams and planks, a timber–concrete composite slab, or a composite steel–concrete slab is usually adopted [1,2,3].

The strengthening of an existing timber slab can be performed in various ways, e.g., transforming it into a wood–concrete composite floor [4,5]. Timber beams can be strengthened by increasing their cross-section height adding glued wooden boards [6] or using externally bonded (EB) or near-surface-mounted (NSM) reinforcement, usually made by steel or fiber-reinforced polymer (FRP) bars and laminates (see, for instance, [7,8,9]). When timber beam ends are degraded, either they are completely removed and reconstructed (see, e.g., [10,11]), the entire beam is replaced, or new timber or steel beams are inserted next to the existing degraded beams to reinforce them and contribute to support the slab.

One of the issues associated with designing these operations is the definition of the contact pressure distribution between the beams and the supporting masonry walls, which is essential to verify that the masonry under the beams (often made by ancient and poor-quality bricks) is able to adequately resist the new applied load [12]. Furthermore, the knowledge of this pressure distribution allows for computing the local stress acting on the timber beam end, verifying its capacity, in relation to the timber compressive strength perpendicular to the grain [13,14,15].

In old masonry structures, it is difficult to perform proper experimental tests providing reliable values of the mechanical properties, also in view of the large heterogeneity that characterizes this material, so that the statistical dispersion of the strength and stiffness parameters may be quite large. Ancient buildings are often the result of different construction events that occurred over time, which makes estimating the masonry properties difficult, also because they often vary considerably in different areas of the structure. Furthermore, historic masonry constructions present quite complex structural schemes that make their analysis cumbersome [16,17,18]. In current design practice, a maximum allowable contact pressure is usually assumed and checked against the pressure transmitted by the supported beam. Although accurate models were proposed in the literature, they are too complex to be adopted in common practice [19]. For this reason, it is crucial to have a fast and reliable method to estimate the contact pressure between the masonry wall and the beam resting on it.

The issue of determining the contact pressure between beams and supporting masonry walls is not properly addressed in the scientific literature, and no simple design formulations, suitable for common practice, are available. In general, no specific calculation is carried out to verify the stress distribution at the beam end, which can result in service and structural issues and reduce the slab and masonry durability. In this paper, a simple approach to compute the support length of beams resting on masonry walls is proposed. First, the approach is explained in detail, providing the exact analytical solution and a simplified analytical formulation suitable for practitioners. Then, the reliability of the method is assessed by comparing the analytical solutions with corresponding solutions obtained by numerical models of various case studies representative of typical applications. Although specific case studies were considered, the assessment can be easily extended to other cases with different material and geometrical properties. The results obtained show that the proposed method allows for a simple and reliable design of the support conditions able to guarantee proper functionality of the structure.

## 2. Study of the Beam-Support Contact Pressure

This paper analyzes the case of a beam laying on a masonry wall. The first step to obtain a simple and reliable formulation for the maximum contact pressure between the beam and wall is the definition of the structural scheme. In this paper, the beam was assumed as simply supported on distributed and independent springs, i.e., the masonry wall was modeled by means of the Winkler spring model [20,21]. Despite being a rather rough idealization of the reality and that more complex methods are available, the Winkler spring approach still constitutes the state-of-practice in many situations, e.g., for the analysis of mat foundations [22]. Furthermore, it was successfully used to model the behavior of masonry-infilled frames where the masonry behavior was described as a Winkler elastic foundation [23].

Investigating the mechanical properties of existing masonry is quite difficult. For instance, the overall elastic modulus can be experimentally determined by means of a double flat jack, but this non-destructive testing method can provide only a local indication of this mechanical parameter, without any information about its spatial variability, unless this technique is widely applied in the structure. Therefore, the adoption of models more complex than the Winkler spring model would not necessarily provide more accurate results since the output will always be affected by the inaccuracy of available input data.

It should be noted that the simply supported beam scheme is generally adopted both in old constructions, where timber beams are supported by masonry walls, and for new or strengthened beams placed in the same positions of the previous or not strengthened ones. The adoption of this structural scheme implies the condition of maximum static effort be represented by the application of the maximum uniformly distributed load (dead, permanent, and variable load). In the case where the variable load is not constant along the beam axis, a uniformly distributed load high enough to provide a bending moment higher than the real bending moment in any section of the beam could be cautiously considered.

**Figure 1:**
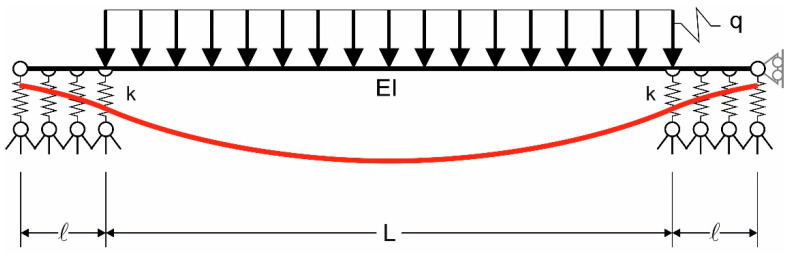
Static scheme adopted.

Therefore, the analysis in this paper will be carried out considering a simply supported beam with a uniformly distributed load, as shown in Figure 1. In Figure 1, q is the total applied load (dead + permanent + variable load) acting on the beam, EI is the beam bending stiffness, k is the stiffness per unit length of the distributed springs, L is the clear span of the beam, and ℓ is the length of the contact (support) area.

**Figure 2:**
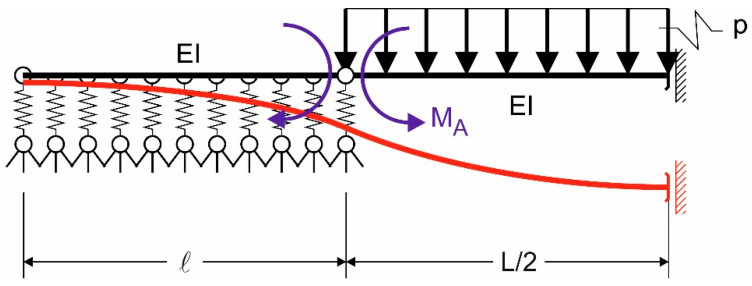
Application of the force method to half of the simply supported beam.

The bending moment acting on the beam in Figure 1 can be obtained by means of the force method (see Figure 2), using the solutions for beams of finite length on the elastic foundation (Winkler spring model) loaded by a concentrated moment at the end [24].

**Figure 3:**
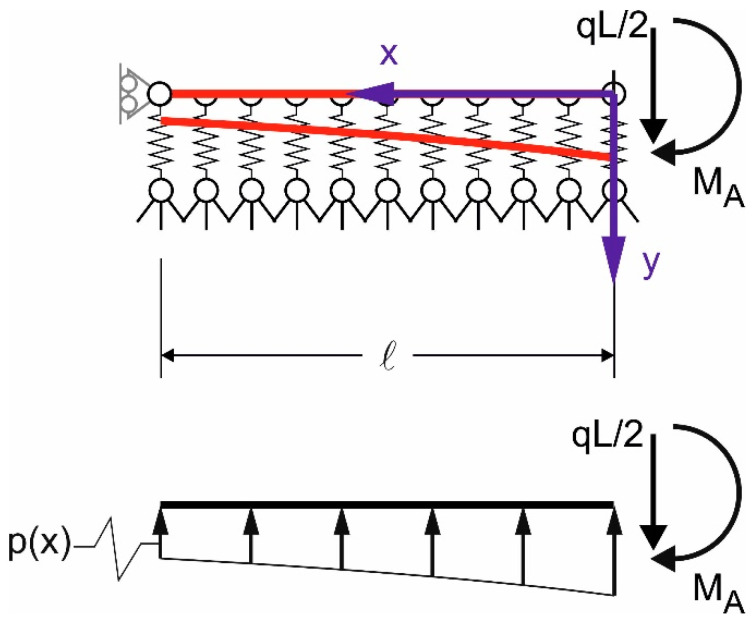
Vertical displacement and contact pressure of the beam end.

The bending moment acting at the beam end, M_A_, is then:

(1) $$M_{A}=\frac{qL}{12\lambda }\frac{\lambda^{2}L^{2}\left[\sinh^{2}\left(\lambda \ell \right)-\sin^{2}\left(\lambda \ell \right)\right]-6\left[\sinh^{2}\left(\lambda \ell \right)+\sin^{2}\left(\lambda \ell \right)\right]}{\lambda L\left[\sinh^{2}\left(\lambda \ell \right)-\sin^{2}\left(\lambda \ell \right)\right]+2\left[\sinh\left(\lambda \ell \right)\cosh\left(\lambda \ell \right)+\sin\left(\lambda \ell \right)\cos\left(\lambda \ell \right)\right]}$$

where $\lambda =\sqrt[{4}]{\frac{k}{4EI}}$. The vertical deflection, y(x), of the beam is:

(2) $$\begin{array}{l} y(x)=\frac{q\lambda L}{k}\frac{1}{\sinh^{2}\left(\lambda \ell \right)-\sin^{2}\left(\lambda \ell \right)}\{\sinh\left(\lambda \ell \right)\cos\left(\lambda x\right)\cosh\left[\lambda \left(\ell -x\right)\right]+\\ -\sin\left(\lambda \ell \right)\cosh\left(\lambda x\right)\cos\left[\lambda \left(\ell -x\right)\right]+\\ -\frac{1}{6}\frac{\lambda^{2}L^{2}\left[\sinh^{2}\left(\lambda \ell \right)-\sin^{2}\left(\lambda \ell \right)\right]-6\left[\sinh^{2}\left(\lambda \ell \right)+\sin^{2}\left(\lambda \ell \right)\right]}{\lambda L\left[\sinh^{2}\left(\lambda \ell \right)-\sin^{2}\left(\lambda \ell \right)\right]+2\left[\sinh\left(\lambda \ell \right)\cosh\left(\lambda \ell \right)+\sin\left(\lambda \ell \right)\cos\left(\lambda \ell \right)\right]}\cdot \\ {}[\sinh\left(\lambda \ell \right)(\cosh\left[\lambda \left(\ell -x\right)\right]\sin\left(\lambda x\right)-\sinh\left[\lambda \left(\ell -x\right)\right]\cos\left(\lambda x\right))+\\ +\sin\left(\lambda \ell \right)\left(\sinh\left(\lambda x\right)\cos\left[\lambda \left(\ell -x\right)\right]-\cosh\left(\lambda x\right)\sin\left[\lambda \left(\ell -x\right)\right]\right)]\} \end{array}$$

where x is the coordinate along the beam longitudinal axis, with origin at the beam end (Figure 3). The contact pressure is p(x) = y(x)k/b, where b is the width of the beam–masonry contact surface.

This solution is valid as long as perfect interaction between the timber beam and the masonry wall is guaranteed along the whole contact length, for example when the beam is embedded in the wall, which is the most frequent configuration in historic masonry buildings with original timber slabs.

**Figure 4:**
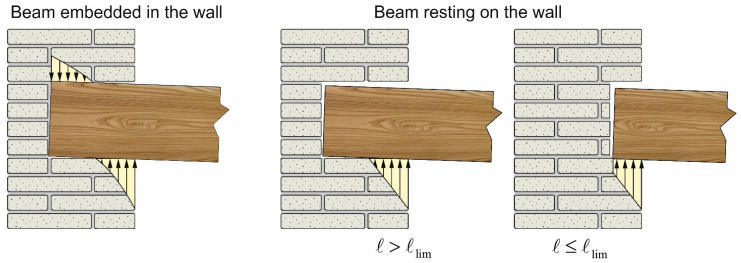
Typical configurations of timber beams resting on masonry walls.

However, in the case of roof slabs, the wall above the beam may be absent. Similarly, when existing timber beams are replaced or strengthened, it is usually preferred to place the new/strengthened beams in the existing seat, avoiding direct contact with masonry to avoid possible thermal bridges (when using steel profiles) and prevent degradation of the timber beam due to possible wall humidity (Figure 4). In all of these cases, the beam is resting on the wall, and Equation (2) applies only when the support area is fully compressed. This condition can be verified by calculating the distance, $\ell_{\lim}$, from the beam end at which the contact pressure becomes nil:

(3) $$\ell_{\lim}=\min\left\{\ell :y(x=\ell )=0\right\}$$

If $\ell \leq \ell_{\lim}$, the solution for the beam resting on the wall is still given by Equation (2). Otherwise, Equation (2) can still be employed substituting ℓ with $\ell_{\lim}$.

The limit length, $\ell_{\lim}$, can be computed enforcing the condition provided by Equation (3) into Equation (2):

(4) $$\begin{array}{l} {y(\ell )|}_{\ell =\ell_{\lim}}=\frac{qL\lambda }{k}\frac{1}{\sinh^{2}\left(\lambda \ell_{\lim}\right)-\sin^{2}\left(\lambda \ell_{\lim}\right)}\{\sinh\left(\lambda \ell_{\lim}\right)\cos\left(\lambda \ell_{\lim}\right)-\sin\left(\lambda \ell_{\lim}\right)\cosh\left(\lambda \ell_{\lim}\right)+\\ -\frac{1}{3}\frac{\lambda^{2}L^{2}\left[\sinh^{2}\left(\lambda \ell_{\lim}\right)-\sin^{2}\left(\lambda \ell_{\lim}\right)\right]-6\left[\sinh^{2}\left(\lambda \ell_{\lim}\right)+\sin^{2}\left(\lambda \ell_{\lim}\right)\right]}{\lambda L\left[\sinh^{2}\left(\lambda \ell_{\lim}\right)-\sin^{2}\left(\lambda \ell_{\lim}\right)\right]+2\left[\sinh\left(\lambda \ell_{\lim}\right)\cosh\left(\lambda \ell_{\lim}\right)+\sin\left(\lambda \ell_{\lim}\right)\cos\left(\lambda \ell_{\lim}\right)\right]}\\ \sinh\left(\lambda \ell_{\lim}\right)\sin\left(\lambda \ell_{\lim}\right)\}=0 \end{array}$$

Equation (4) can be made dimensionless setting:

(5) $$\begin{array}{l} \delta =\lambda L=\sqrt[{4}]{\frac{kL^{4}}{4EI}}\\ \beta_{\lim}=\frac{\ell_{\lim}}{L} \end{array}$$

Which provides:

(6) $$\begin{array}{l} \sinh\left(\delta \beta_{\lim}\right)\cos\left(\delta \beta_{\lim}\right)-\sin\left(\delta \beta_{\lim}\right)\cdot \cosh\left(\delta \beta_{\lim}\right)+\\ -\frac{1}{3}\sinh\left(\delta \beta_{\lim}\right)\sin\left(\delta \beta_{\lim}\right)\\ \frac{\delta^{2}\left[\sinh^{2}\left(\delta \beta_{\lim}\right)-\sin^{2}\left(\delta \beta_{\lim}\right)\right]-6\left[\sinh^{2}\left(\delta \beta_{\lim}\right)+\sin^{2}\left(\delta \beta_{\lim}\right)\right]}{\delta \left[\sinh^{2}\left(\delta \beta_{\lim}\right)-\sin^{2}\left(\delta \beta_{\lim}\right)\right]+2\left[\sinh\left(\delta \beta_{\lim}\right)\cosh\left(\delta \beta_{\lim}\right)+\sin\left(\delta \beta_{\lim}\right)\cos\left(\delta \beta_{\lim}\right)\right]}=0 \end{array}$$

The solution of Equation (6) can be obtained iteratively. Values of the normalized limit length, β_lim_, for different values of the normalized length, δ, are provided in Table 1. For instance, the value of β_lim_ for δ = 32.5 can be found in Table 1 in the intersection cell of the column of tens named 30 and of the row of units named 2.5, which provides 0.002321.

As can be noticed from Equation (6), β_lim_ does not depend on the applied load q, but only on the characteristics of the beam and masonry support. 

**Figure 5:**
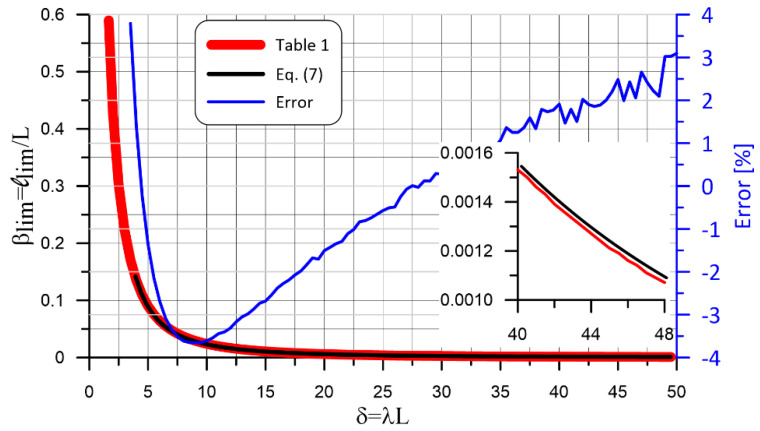
Comparison between results in Table 1 and Equation (7).

The relationship between β_lim_ and λL described by Equation (6) (see Table 1) is depicted in Figure 5. For values of 3.5 ≤ δ ≤ 50, Equation (6) can be accurately approximated by Equation (7) as:

(7) $$\beta_{\lim}=2.023\cdot \delta^{-1.943}$$

The relationship between β_lim_ and λL provided by Equation (7) is represented in Figure 5 along with the error made using the approximated result of Equation (7) with respect to the exact solution of Equation (6). In Figure 5, a zoom-in of the results for 40 ≤ λL ≤ 48 is also provided to show the small difference between the two solutions depicted. 

It should be noted that the range 3.5 ≤ δ ≤ 0 is representative of usual practical applications for both timber and steel beams. Indeed, considering a masonry elastic modulus, Em, in the range 700 MPa ≤ Em ≤ 7000 MPa [25], the stiffness per unit length of the distributed springs, k, varies in the range 2400 MPa ≤ k ≤ 7200 MPa (see Equation (13) below). This range of validity was obtained considering a ratio between the beam span, L, and cross-sectional height, h, within 5 ≤ L/h ≤ 20, since values higher than 20 lead to steel and timber beams too deformable for practical applications. Furthermore, the elastic modulus, E, of timber beams is usually in the range 7000 MPa ≤ E ≤ 16,000 MPa, and the second moment of area can be considered equal to I = h4/12, since the square cross-section is associated with the least waste starting from a circular trunk and producing a rectangular cross-section. In the case of steel beams (elastic modulus Es = 200 GPa), I = ψh4, where 0.012 ≤ ψ ≤ 0.06 when adopting European I (IPE) or wide-flange (HE) steel beams.

**Figure 6:**
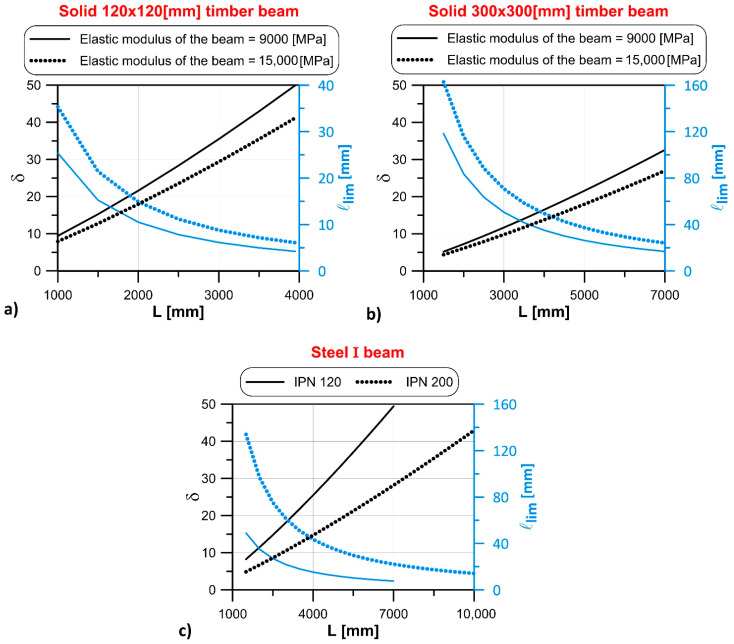
Variation of the normalized length, δ, and limit length, $\ell_{\lim}$, with the span, L, for (**a**) 120 mm × 120 mm and (**b**) 300 mm × 300 mm cross-section timber beams and (**c**) steel IPN beams.

The variations of the normalized length, δ, and limit length, $\ell_{\lim}$, with respect to the span, L, obtained solving Equation (6), are depicted in Figure 6. Figure 6a shows that, considering a solid timber beam with a 120 mm × 120 mm cross-section and elastic modulus of either 9000 or 15,000 MPa, the normalized length, δ, increases with increasing L, whereas the limit length, $\ell_{\lim}$, shows an opposite trend. Given a certain L, the increase in the elastic modulus is responsible for an increase of δ and for a corresponding decrease of $\ell_{\lim}$. The same behavior can be observed in Figure 6b, where a solid timber beam with a 300 mm × 300 mm cross-section and elastic modulus of either 9000 or 15,000 MPa was considered. Analogously, considering a steel IPN beam with a height equal to 120 or 200 mm (Figure 6c), the normalized length, δ, increases with increasing L, while $\ell_{\lim}$ decreases. Figure 6 provides an indication of the effect of the beam geometrical and mechanical properties on the obtained normalized and limit lengths. In Section 4, a validation of the results obtained by Equation (6) is provided considering representative cases of solid and laminated timber beams and of steel beams resting on masonry walls. 

## 3. Estimation of the Spring Stiffness

**Figure 7:**
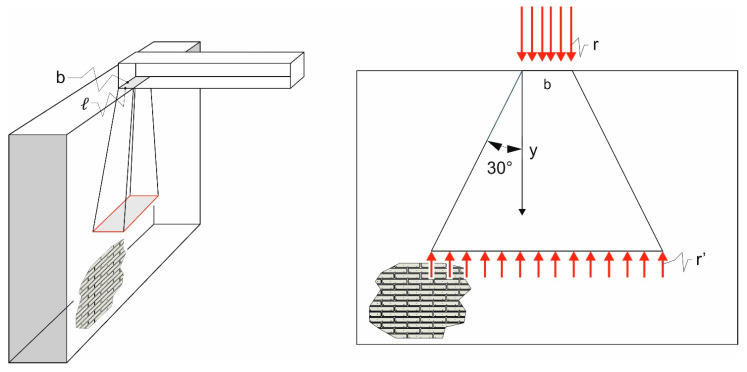
Stress distribution assumed in the supporting wall.

Equations (6) and (7) require the knowledge of the spring stiffness that simulates the mechanical behavior of the masonry wall. The estimation of this spring stiffness is a cumbersome task, since it is affected by the stress redistribution in the masonry due to the load applied by the beam end. Assuming that the load applied by the beam end is transmitted to the masonry supporting wall following a linear path with inclination equal to 30° with respect to the applied load direction (Figure 7) [26,27,28,29], the stress, r’(y), at a distance, y, from the contact surface can be written as:

(8) $$r^{\prime }(y)=r\frac{b\ell }{\left[b+2y\tan\left(30{}^{\circ}\right)\right]\left[\ell +y\tan\left(30{}^{\circ}\right)\right]}$$

where r is the mean value of the pressure on the contact surface. A linear distribution of the applied load within the masonry (Figure 7) is based on the assumption that no openings that can alter the stress distribution are located close to the beam, and that it is located sufficiently far from the masonry edge. Future studies considering masonry walls with openings will provide indications on the variation of the support length when the masonry characteristics below the applied load cannot be considered constant.

The distance, h, between the point of application of the load and the masonry cross-section, where the stress, r’, attains 0.01r, can be computed from Equation (8), setting r’(h) = 0.01r:

(9) $$r\frac{b\ell }{\left[b+2h\tan\left(30{}^{\circ}\right)\right]\left[\ell +h\tan\left(30{}^{\circ}\right)\right]}=0.01r$$

Which provides:

(10) $$h=\frac{\sqrt{{\left(b+2\ell \right)}^{2}+792b\ell }-\left(b+2\ell \right)}{4\tan\left(30{}^{\circ}\right)}$$

The mean vertical displacement of the contact surface can then be computed:

(11) $$\begin{array}{l} \Delta h=\int_{0}^{h}\frac{r^{\prime }(y)}{E_{m}}dy=\\ =\frac{rb\ell }{E_{m}}\left(\frac{1}{-2\ell +b}\right)\left[\frac{1}{\tan\left(30{}^{\circ}\right)}\right]\left\{\ln\left[\ell +h\tan\left(30{}^{\circ}\right)\right]-\ln\left[b+2h\tan\left(30{}^{\circ}\right)\right]+\ln\left(\ell \right)-\ln\left(b\right)\right\} \end{array}$$

where E_m_ is the masonry elastic modulus along the y-direction. Values of E_m_ for various types of ancient and modern masonry can be found in [25,30,31] or can be obtained by destructive and non-destructive tests of the studied masonry. The spring stiffness, k, can then be obtained as:

(12) $$k=\frac{r\cdot b}{\Delta h}$$

(13) $$k=\frac{E_{m}\left(b-2\ell \right)\tan\left(30{}^{\circ}\right)}{\ell \left\{\ln\left[\ell +h\tan\left(30{}^{\circ}\right)\right]+\ln\left(b\right)-\ln\left[b+2h\tan\left(30{}^{\circ}\right)\right]-\ln\left(\ell \right)\right\}}$$

Equation (10), and therefore Equation (13), applies when $\ell \leq \ell_{\lim}$. When $\ell \geq \ell_{\lim}$, Equations (10) and (13) can still be employed replacing ℓ with $\ell_{\lim}$. This approach can be also adopted to estimate the spring stiffness when the beam is embedded in the wall (see Figure 4). It should be noted that, since $\ell_{\lim}$ depends on k, the following steps are required:

- Calculate $\ell_{\lim}$ and the corresponding ${k|}_{\ell =\ell_{\lim}}$ (iteratively solving Equations (6) and (13), e.g., with the fixed-point iteration method).
- Compare ℓ with $\ell_{\lim}$. If $\ell <\ell_{\lim}$, compute k considering ℓ in Equation (13); otherwise, ${k=k|}_{\ell =\ell_{\lim}}$.

In rehabilitation interventions, a rubber bearing pad is often interposed between the beam and masonry. When a rubber pad is used, the spring stiffness, k, can be evaluated combining the stiffness of the two materials (rubber pad and masonry) in series:

(14) $$\frac{1}{k}=\frac{1}{k_{w}}+\frac{1}{k_{p}}=\frac{k_{w}+k_{p}}{k_{w}k_{p}}\Rightarrow \;k=\frac{k_{w}k_{p}}{k_{w}+k_{p}}$$

where k_w_ is the stiffness of the wall computed by Equation (13) and k_p_ is the stiffness of the rubber pad. k_p_ can be computed considering no pressure diffusion across the rubber pad thickness, i.e., the pressure acting on the two opposite faces of the pad are equal, and assuming that transverse expansion is prevented by the presence of friction with the masonry and beam:

(15) $$k_{p}=\frac{b_{p}}{h_{p}}\frac{E_{p}}{1-\nu_{p}^{2}}$$

where E_p_ is the elastic modulus of the rubber, ν_p_ is its Poisson’s ratio, and b_p_ (b_p_ ≤ b) and h_p_ are the width and height of the pad, respectively. Typical values for the parameters considered are:

(16) $$\begin{array}{l} k_{w}=\mathrm{from}\;2400\;\mathrm{to}\;7200\;{[N/\mathrm{mm}}^{2}]\\ E_{p}≃2.5\;{N/\mathrm{mm}}^{2}\\ \nu_{p}≃0.39\\ h_{p}=\mathrm{from}\;10\;\mathrm{to}\;25\;\left[\mathrm{mm}\right]\\ b_{p}=\mathrm{from}\;50\;\mathrm{to}\;200\;\left[\mathrm{mm}\right] \end{array}$$

Considering the values in Equation (16) provides k_p_ ≤ 0.01 k_w_. Therefore:

(17) $$k=\frac{k_{w}k_{p}}{k_{w}+k_{p}}≃k_{p}$$

## 4. Finite Element Modeling

In this section, finite element models will be used to validate the analytical formula proposed in the previous sections. The commercial software Abaqus [32] was used to model different types of beams resting on masonry walls. 

Numerical modeling of timber elements still represents a complex and difficult issue, due to the different behavior that timber presents under axial tensile (elasto-brittle) and compressive (elasto-plastic) stress states, as well as to its natural anisotropy, both in the linear and nonlinear regime. Therefore, the bending response of a timber beam up to failure cannot be described by a linear-elastic model, but it must take into account a ductile compressive behaviour that leads to the formation of a ductile region in the stress–strain response [33,34,35]. 

To accurately represent the behavior of timber in the nonlinear phase, it is necessary to model the material considering its orthotropic and asymmetric behavior under normal stresses. However, since the approach presented in Section 2 and Section 3 is based on the assumption that under service conditions the contact pressure does not induce damage to the members in contact, the beam and wall are assumed to have a linear elastic behavior. Although complex non-linear numerical models can be considered to describe the masonry behavior, since the structure response under service conditions should remain elastic, the numerical analysis did not consider the inelastic behavior of materials. Possible cracking of masonry due to the application of the maximum service load could be accounted for using an elastic model with a reduced masonry stiffness. The occurrence of damage to the masonry wall or to the supported beam, e.g., as a result of the application of exceptional or ultimate loads, would affect the masonry–beam interaction. The variation and evolution of the masonry–beam contact pressure due to the occurrence of damage could be studied considering complex masonry and beam constitutive laws able to account for their non-linear behavior. However, the description of the masonry–beam interaction under ultimate loads is out of the scope of this study, which is focused on the structural behavior under service conditions. 

In the numerical models, the beam geometry and material properties are varied to represent different types of beams (timber and steel), while masonry is modeled with dimensions of 6.0 m (width) × 5.0 m (height) × 0.5 m (depth), and a hard contact (no overclosure [32]) is considered between the beam and masonry, neglecting the presence of interface friction. After a convergence study to investigate the effect of mesh size and element type on the numerical results, eight-node solid elements having an edge of approximately 10 mm in correspondence of the contact area and larger dimensions far from the contact area were selected. Two main cases were considered: (a) direct support, where the beam was directly supported by the masonry, and (b) indirect support, where a rubber pad was inserted between the beam and masonry. 

### 4.1. Direct Support

#### 4.1.1. Timber Beams

**Figure 8:**
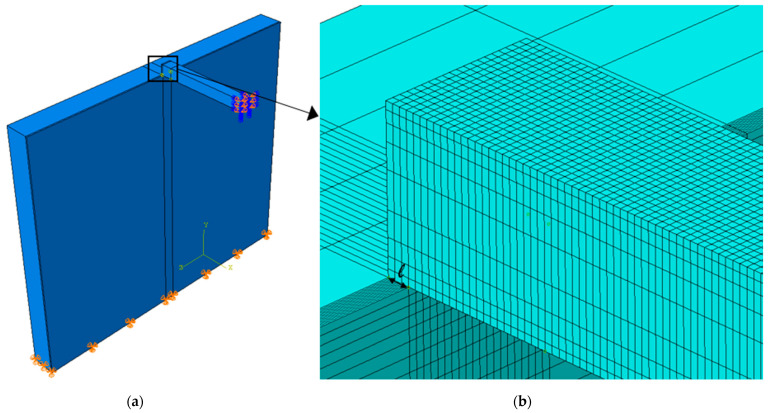
(**a**) Finite element model of a timber beam resting on a masonry wall and (**b**) detail of the contact portion.

The first case analyzed is a timber beam directly supported by a masonry wall. Two different geometries were considered: a 200 mm × 200 mm solid timber beam and an 80 mm × 266 mm laminated timber beam. In both cases, L = 3600 mm and E_m_ = 2400 MPa. A uniform load of 4.0 kN/m was applied on the beam. Figure 8 shows the finite element model of the solid timber beam–masonry system, where only half of the beam was modeled, exploiting its symmetry. The masonry base was restrained from movement in any direction, while rotations were allowed.

The material properties adopted for the two beams were taken from [35,36], respectively, and are reported in Table 2. In the numerical models, the support length, ℓ, was varied. Based on the results of the analytical approach, ℓ = 30 mm and ℓ = 10 mm were considered to investigate the effect of a support length higher and lower than the limit length, respectively, on the distribution of the beam–masonry contact pressure.

**Figure 9:**
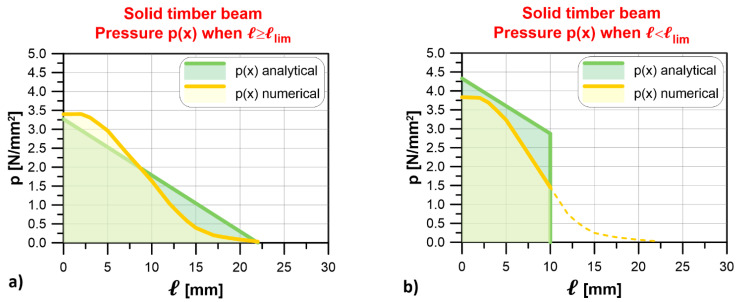
Solid timber beam: comparison of contact pressure profiles at the beam–wall interface obtained with the analytical approach and numerical model for (**a**) $\ell \geq \ell_{\lim}$ and (**b**) $\ell <\ell_{\lim}$.

Figure 9 shows the comparison between the contact pressure profiles along the mid-width beam–wall interface obtained by the analytical approach and the numerical model with the solid timber beam. The numerical contact pressure was defined as the stress in the direction of the applied load induced on the masonry by the resting beam. In Figure 9a, the support length, ℓ = 30 mm, is higher than the limit length, $\ell_{\lim}$ = 21.95 mm, obtained by the analytical approach, which also provided k = 7298 MPa. In this case, the limit length obtained by the numerical model, defined as the distance between the beginning of the support length and the point where the contact pressure attains a null value (Figure 9), was $\ell_{\lim}$ = 21.52 mm, which confirms the accuracy of the analytical approach proposed (1.96% difference). 

Figure 9b shows the analytical and numerical contact pressure profiles for a support length ℓ = 10 mm. In this case, analytical and numerical results are similar. However, in the numerical model, the contact pressure diffuses in the masonry wall beyond the end of the beam–wall interface area, as shown by the dashed line in Figure 9b, which explains why the numerical contact pressure is lower than the corresponding analytical result along ℓ.

**Figure 10:**
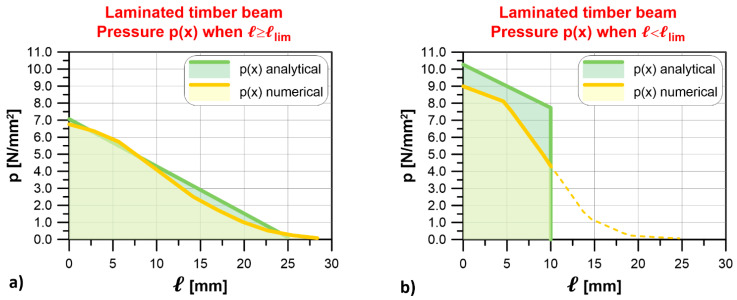
Laminated timber beam: comparison of contact pressure profiles at the beam–wall interface obtained with the analytical approach and numerical model for (**a**) $\ell \geq \ell_{\lim}$ and (**b**) $\ell <\ell_{\lim}$.

The contact pressure profiles obtained with the laminated timber beam–masonry model are shown in Figure 10. The numerical and analytical profiles obtained for a support length ℓ = 30 mm, which is higher than the limit length provided by the analytical approach, $\ell_{\lim}$ = 25.24 mm (k = 3912 MPa), are quite similar (Figure 10a). Indeed, the limit length estimated by the numerical model was $\ell_{\lim}$ = 28.24 mm, with only 11.89% difference. As in the case of the solid timber beam–masonry model, considering a support length ℓ = 10 mm, lower than the limit length, the numerical contact pressure propagated beyond the contact area (Figure 10b). Therefore, the numerical contact pressure profile slightly underestimated that provided by the analytical model for ℓ = 10 mm.

#### 4.1.2. Steel Beams

Frequently, slabs of historical masonry buildings were realized using steel beams (see, e.g., [37,38,39,40]). To assess the accuracy of the proposed analytical formulation in the case of steel beams resting on masonry walls, a numerical model comprising an IPN220 steel beam resting on a masonry wall was realized. The steel beam was modeled as a linear elastic material with elastic modulus E_s_ = 200 GPa and Poisson’s ratio ν = 0.2. Due to the symmetry, only half of the beam was modeled (L = 3600 mm) and a uniform load of 4.0 kN/m was applied on top of it.

Three different support lengths were considered: (a) ℓ = 80 mm, (b) ℓ = 30 mm, and (c) ℓ = 120 mm. Since the analytical limit length is $\ell_{\lim}$ = 59.93 mm (k = 2789 MPa), the former case (a) represents a situation where the support length is greater than the limit length (namely, $\ell \geq \ell_{\lim}$), while the second case (b) represents a situation where the support length is lower than $\ell_{\lim}$. In case (c), the support length considered entails for the presence of tensile stresses at the beam–masonry interface. The stresses, which would not be present if the beam was laying on the masonry wall, can be assumed to have the same effect of the compressive stresses exerted on the extrados of the beam when it is embedded in the masonry wall (see Figure 4). Therefore, case (c) represents an IPN220 steel beam embedded within the masonry wall. 

**Figure 11:**
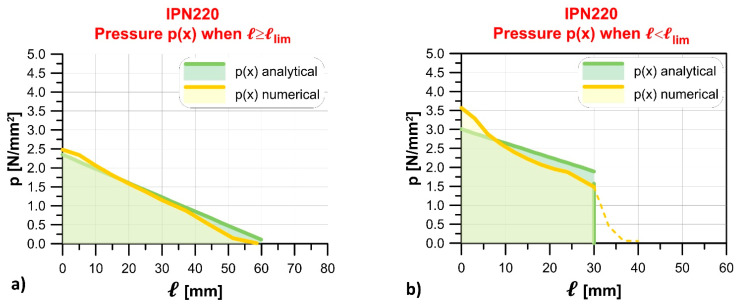

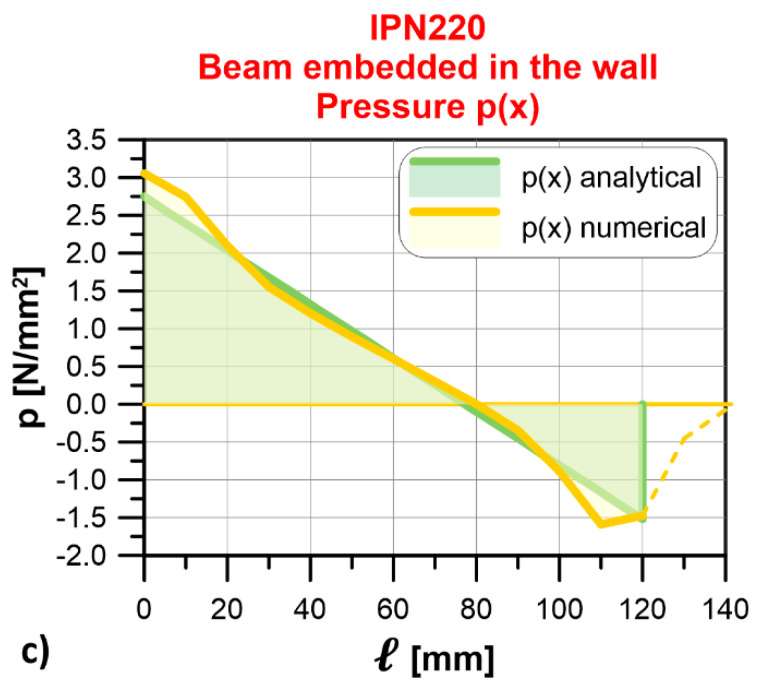
Steel IPN220 beam: comparison of contact pressure profiles at the beam–wall interface obtained with the analytical approach and numerical model for (**a**) $\ell \geq \ell_{\lim}$, (**b**) $\ell <\ell_{\lim}$, and (**c**) beam embedded in the wall.

Figure 11 shows the comparison between analytical and numerical results obtained for each case. The analytical and numerical contact pressure profiles are in good agreement both when $\ell \geq \ell_{\lim}$ (case a) and when the beam is embedded within the masonry wall (case c). In the latter case, the numerical profile propagated beyond the contact area (see dashed line in Figure 11c). Furthermore, the limit length obtained numerically, $\ell_{\lim}$ = 59.39 mm, was approximately equal to the limit length estimated analytically in case (a). When $\ell <\ell_{\lim}$ (case b), the analytical solution provided a contact pressure at the end of the masonry–beam interface slightly lower than that obtained by the numerical model. Furthermore, in the numerical model, the contact stress also distributed beyond the support length (see dashed line in Figure 11c). The differences observed, however, appear limited and confirm the reliability of the analytical approach in estimating the limit length and contact pressure distribution.

### 4.2. Indirect Support

**Figure 12:**
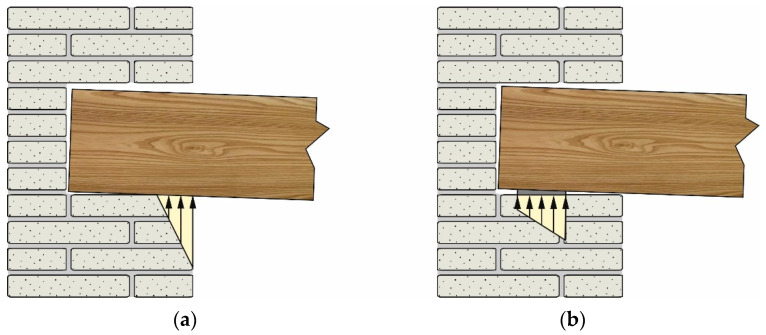
Qualitative contact pressure distributions in the case of (**a**) direct and (**b**) indirect support.

Section 4.1 showed that the limit length of timber beams resting on masonry walls may be quite small. This might lead to problems of local crushing of masonry, which can be solved by inserting a rubber pad under the beam to:

1. Move the resultant of the contact pressure distribution towards the center of the masonry wall (Figure 12).
2. Distribute the contact pressure over a higher length with respect to the case of direct support (the presence of a rubber pad reduces k, λ, and δ, which is equivalent to increasing the flexibility of the support, thus increasing β_lim_ compared to the case of direct support).
3. Provide thermohygrometric insulation of the beam by completely eliminating the direct contact with the masonry.

According to the analytical approach provided in Section 3, when a rubber pad is inserted between the masonry wall and the beam (typically a timber beam) to control the contact pressure between these two structural elements, the length of the contact area strictly depends on the stiffness of the neoprene pad, which is usually quite low. According to Equation (15) and assuming the values reported in Equation (16), for a rubber pad with width b_p_ = 200 mm and thickness h_p_ = 20 mm, the stiffness is:

(18) $$k_{p}=\frac{b_{p}}{h_{p}}\frac{E_{p}}{1-\nu_{p}^{2}}=\frac{200\;}{20\;}\frac{2.5}{1-0.39^{2}}=29.48\;\mathrm{MPa}$$

In the case of the solid timber beam, the spring stiffness, k, is (see Equation (17)):

(19) $$\begin{array}{c} k_{w}=7392{\;N/\mathrm{mm}}^{2}\\ k_{p}=29.48{\;N/\mathrm{mm}}^{2} \end{array}\;\Rightarrow \;k=29.48\;{N/\mathrm{mm}}^{2}\;≃k_{p}$$

and $\ell_{\lim}$ becomes (see Equation (7)):

(20) $$\ell_{\lim}=1.8L{\left(\frac{kL^{4}}{4EI}\right)}^{-0.475}=1.8\cdot 3600\;{\left(\frac{29.48\cdot 3600^{4}}{4\cdot 14500\cdot 133,333,333.3}\right)}^{-0.475}=300.99\;\mathrm{mm}$$

**Figure 13:**
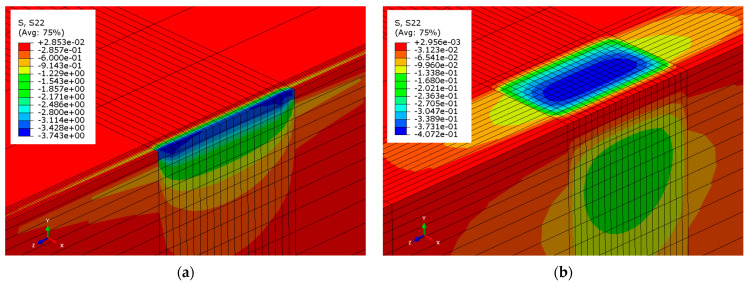
Contact pressure contours on the masonry surface of solid timber beam–masonry models (**a**) without and (**b**) with rubber pad.

This calculation shows that the limit length obtained with the rubber pad ($\ell_{\lim}$ = 300.99 mm) is much higher than that determined for direct contact ($\ell_{\lim}$ = 21.95 mm). Therefore, values of contact pressure at the beam–masonry interface decrease significantly. This result was confirmed by inserting a rubber pad in the solid timber beam–masonry numerical model (Section 4.1.1). Figure 13 shows the pressure contours on the masonry surface when the support length is exactly equal to the numerical solid timber beam–masonry limit length, $\ell_{\lim}$ = 21.52 mm (Figure 13a), and when a rectangular rubber pad with length 100 mm, width 200 mm, and the properties introduced above is inserted below the beam (Figure 13b). Adopting Equation (2), the contact pressure under the rubber pad varies from 0.39 to 0.33 MPa. This small variation is confirmed by the numerical model, where the contact pressure varies between 0.41 MPa and 0.28 MPa.

When considering the laminated timber beam of Section 4.1.1, the spring stiffness, k, is (according to Equation (17) and considering a rubber pad with width b_p_ = 80 mm and thickness h_p_ = 20 mm):

(21) $$\begin{array}{l} k_{w}=3677.6\;{N/\mathrm{mm}}^{2}\\ k_{p}=11.79\;{N/\mathrm{mm}}^{2} \end{array}\;\Rightarrow \;k=\frac{k_{w}k_{p}}{k_{w}+k_{p}}=11.75\;{N/\mathrm{mm}}^{2}≃k_{p}$$

Which provides (see Equation (7)):

(22) $$\ell_{\lim}=1.8L{\left(\frac{kL^{4}}{4EI}\right)}^{-0.475}=1.8\cdot 3600\cdot {\left(\frac{11.75\cdot 3600^{4}}{4\cdot 11080\cdot 125,473,973.3}\right)}^{-0.475}=398.26\;\mathrm{mm}$$

**Figure 14:**
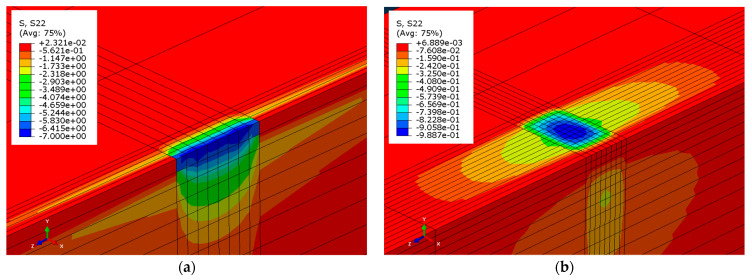
Contact pressure contours on the masonry surface of laminated timber beam–masonry models (**a**) without and (**b**) with rubber pad.

Therefore, also in this case, the presence of the rubber pad markedly increases the contact area and significantly reduces the maximum contact pressure, as confirmed by the comparison with the results of the same laminated timber beam–masonry model of Section 4.1.1. Namely, the contours of the masonry contact pressure for a support length equal to the numerical laminated beam–masonry limit length, $\ell_{\lim}$ = 28.24 mm, are shown in Figure 14a, while the corresponding masonry contact pressure obtained by the same model, where a rubber pad with length 100 mm, width 80 mm, and the properties considered in this section was inserted beneath the beam, are shown in Figure 14b. 

The contact pressure obtained analytically along the rubber pad length (see Equation (2)) varied from 0.95 MPa to 0.86 MPa, whereas that obtained by the numerical model varied between 0.49 MPa and 0.99 MPa. 

The same analysis carried out for solid and laminated timber beams was performed for the IPN220 steel beam discussed in Section 4.1.2. The results obtained confirmed the observations made for timber beams and are not provided here for the sake of brevity. The comparison between analytical and numerical results discussed in this section confirmed the reliability of the analytical procedure proposed for different types of beam and configuration.

## 5. Conclusions

An analytical solution that allows to assess the support length of beams resting on masonry walls through the calculation of the contact pressure distribution between a timber (or steel) beam and a masonry wall, with or without the interposition of a rubber pad, was presented in this paper. The knowledge of the beam–masonry interface stress distribution allows for guaranteeing the integrity of the wall, as well as of timber beams, for which otherwise it would not be possible to exclude a priori crushing in the direction transversal to the fibers. The analytical solution was obtained assuming a simply supported beam resting on a masonry wall modeled by means of the Winkler spring model. First, the exact solution of the problem was obtained and discussed. Then, a simplified yet accurate approximate analytical solution was provided and validated against the exact solution and then adopted for comparison with corresponding numerical solutions of different representative case studies. 

Different types of beams and configurations were considered, namely solid and laminated timber beams and steel beams that were:

- Embedded in the wall ($\ell >>\ell_{\lim}$).
- Resting on the wall, direct support, $\ell \leq \ell_{\lim}$.
- Resting on the wall, direct support, $\ell >\ell_{\lim}$.
- Resting on the wall, indirect support.

The results obtained and the discussion provided showed that the proposed approach is simple and reliable. Indeed, the use of the approximated analytical solution provided negligible errors with respect to the exact analytical solution. Furthermore, the pressure distributions obtained by numerical models were quite similar to those provided by the analytical solutions, which confirmed the accuracy of the proposed method. 

The adoption of a simple approximated analytical solution or of tabulated solutions make the proposed method suitable for professional practice. Indeed, in each case study considered, the contact pressure distribution was easily obtained by a spreadsheet or a brief computer program (for reference, a possible Visual Basic subroutine that computes $\ell_{\lim}$ is provided in Appendix A). Future developments of this research will include modeling of the masonry–beam interface under ultimate or exceptional loads, which may induce damage to materials affecting the support conditions. Furthermore, studies considering masonry with inhomogeneous characteristics, such as walls with openings, will provide indications on the variation of the support length when the masonry characteristics are not constant. 

## Figures and Tables

**Table 1:** Values of β_lim_ as δ varies.

δ		Tens				
		0	10	20	30	40
units	0.0	---	0.023931	0.006091	0.002721	0.001531
	0.5	---	0.021751	0.005801	0.002631	0.001501
	1.0	---	0.019851	0.005531	0.002551	0.001461
	1.5	0.658521	0.018201	0.005281	0.002471	0.001431
	2.0	0.429331	0.016741	0.005041	0.002391	0.001391
	2.5	0.301531	0.015441	0.004821	0.002321	0.001361
	3.0	0.222781	0.014291	0.004611	0.002251	0.001331
	3.5	0.170881	0.013271	0.004421	0.002181	0.001301
	4.0	0.134951	0.012351	0.004241	0.002121	0.001271
	4.5	0.109101	0.011521	0.004071	0.002061	0.001241
	5.0	0.089921	0.010781	0.003911	0.002001	0.001211
	5.5	0.075321	0.010101	0.003761	0.001941	0.001191
	6.0	0.063961	0.009481	0.003621	0.001891	0.001161
	6.5	0.054971	0.008921	0.003481	0.001841	0.001141
	7.0	0.047721	0.008411	0.003351	0.001791	0.001111
	7.5	0.041811	0.007941	0.003231	0.001741	0.001091
	8.0	0.036921	0.007511	0.003121	0.001701	0.001071
	8.5	0.032831	0.007111	0.003011	0.001651	0.001051
	9.0	0.029381	0.006741	0.002911	0.001611	0.001021
	9.5	0.026451	0.006411	0.002811	0.001571	0.001001

E.g., δ = 32.5 ⇒ β_lim_ = 0.002321.

**Table 2:** Elastic moduli adopted to describe the orthotropic elastic behavior of timber.

**Solid Timber Beam**		
Normal Moduli	Poisson’s Ratios	Tangential Moduli
E_1_ = 14.50 GPa	ν_12_ = 0.37	G_1_ = 590 MPa
E_2_ = 1.20 GPa	ν_13_ = 0.43	G_2_ = 590 MPa
E_3_ = 1.20 GPa	ν_23_ = 0.45	G_3_ = 73 MPa
**Laminated Timber Beam**		
Normal Moduli	Poisson’s Ratios	Tangential Moduli
E_1_ = 11.08 GPa	ν_12_ = 0.37	G_1_ = 791 MPa
E_2_ = 0.89 GPa	ν_13_ = 0.42	G_2_ = 744 MPa
E_3_ = 0.55 GPa	ν_23_ = 0.47	G_3_ = 79 MPa

## Data Availability

The data presented in this study are available on request from the corresponding author.

## Appendix A

Input and output data explanation:

EJ = EJ

L = L

Litalic = ℓ

b = b

Em = E_m_

kn = −1 for direct contact, otherwise kn = assigned value

llim = $\ell_{\lim}$

k = k

h = h

Sub Solve (EJ as Double, L as Double, litalic as Double, b as Double, Em as Double, kn as Double, ByRef llim as Double, ByRef k as Double, ByRef h as Double)

Dim Delta as Double

Dim Test, Oldllim as Double

Dim Counter as Integer

‘Initialization:

Counter = 0

llim = litalic

‘Calculation of llim

NewStep:

h = (Sqrt((b + 2*llim)^2 + 792*b*llim) − (b + 2*llim))/2.3094

if kn < 0 then

k = Em*(b − 2*llim)*0.57735/llim/(Log(llim + h*0.57735) + Log(b) − Log(b + 2*h*0.57735) − Log(llim))

Else

k = kn

end

Delta = (k/4/EJ)^0.25*L

Oldllim = llim

llim = L*2.023*Delta^(−1.943)

Test = Abs((llim − Oldllim)/llim)

Counter = Counter + 1

If Counter > 1000 then go to Fail

If Test > 0.0001 then go to New Step

Exit

Fail:

llim = −100

Exit

End Sub
